# Should we digitalize the neurological examination?

**DOI:** 10.1007/s10072-025-08398-y

**Published:** 2025-08-09

**Authors:** Bruno Kusznir Vitturi, Walter Maetzler

**Affiliations:** Department of Neurology, University Hospital Schleswig-Holstein and Kiel University, Kiel, Germany

It is impossible to understand the history of neurology in isolation from the importance of the neurological examination (NE). A large part of the contribution of famous neurologists lies in the discovery of NE tests and maneuvers that are still used, studied and taught today. It is the most readily available part of neurological art to detect health deficits and diagnose a neurological disease. Previously proposed clinical pathways have granted the interpretation of findings extracted from the NE a pivotal role in the diagnostic work-up, making it a key component of the diagnostic process of almost all neurological conditions and a gatekeeper to expensive investigations. For example, Parkinson disease can be diagnosed based only on specific observations extracted from the NE (in combination with anamnesis). For stroke and multiple sclerosis, the NE serves as the primary gatekeeper, guiding the subsequent diagnostic procedures. Few of us neurologists will disagree that NE deserves a central place in our daily work and that we need to train and develop it appropriately.

Especially in an era of digitalization and disruptive new analysis methods, it can therefore make sense to put the state of our NE to the test. There is evidence that advances over the last two decades in areas such as neurophysiology, neuroimaging, neurogenetics, laboratory diagnostics and neuropathology outweigh traditional history taking and physical examination. More worryingly, the quality of NE appears to have been declining during recent decades, and poorer skills contribute to medical errors and adverse events. Reasons postulated for this shift in approach, in addition to improvements in technology in the above-mentioned ‘non-NE’ diagnostic areas, are constraints in time and skills resources. There may be some justification for this: the quality and quantity of training in clinical skills appears to have declined over the past centuries, with deficiencies that begin in medical school and continue through residency and into practice [1]. Only the minority of medical students is observed by a faculty member while performing physical examinations [2]. During the residency, many medical doctors are practically incapable of detecting neurological signs with the necessary accuracy [3]. The inevitable result is the disappearance of the physical examination from consultations with specialized and experienced doctors [4].

We can therefore see here that digital and technical developments have substantially advanced medicine in many areas. Surprisingly, however, it has had the least/most negative effect on the most important examination for neurologists, the NE. The value of NE should be enhanced in our everyday clinical practice. Our patients should be carefully examined (again) and our junior staff should be trained to the highest standards in the performance of NE. Moreover, we claim that the implementation of digital technology and novel AI-based analysis techniques can enable further development and improvement of the NE, which will ultimately benefit the patient of tomorrow, especially from the perspective of increasing frequencies of long disease courses, and the implementation of shared decision making and personalized medicine approaches in our clinical routine. A plan for the development of a digital NE has yet to be developed. However, we can imagine that, for example, with the use of two synchronized smartphone cameras, a room microphone and an eye tracker, the majority of the NE routinely performed in clinics and practices could be analysed digitally and quantitatively.

No digital tool has yet made it into our NE. But there are already examples in the literature that suggest that this could work. For instance, an eye tracker can identify digital biomarkers of the prodromal stage of alfa-synucleinopathies in a low resource setting [5]. Some digital devices, such as pupillometers, can be easily implemented in the daily routine and are not time-consuming [5]. The use of novel analysis approaches is also promising. Several posturography measures were able to discriminate controls from patients with pre-manifest HD, for example [6]. Quantitative analyses performed based on the digital data of the motor function of patients with neurological diseases have the potential to be highly superior to traditional clinical scores to identify disability. An improvement in the quality of the NE may also be achieved in the areas of reporting and comparability of examinations. This is primarily possible because the data can be easily stored and compared with each other in repeated examinations.

The implementation of digital technology and new methods of analysis has, in our opinion, the potential to improve the quality of NE and increase its relevance in the management of our neurological patients on levels beyond the simple examination per se, as mentioned above. First, it can improve the training of examiners and trainees: Digital devices can serve as a means to control the accuracy and quality of clinically obtained information, allowing medical trainees to use them as self-learning tools for neurological examinations. Second, it can improve the quality of disease management in neurologically less well-served regions. As the medical training pathway to acquire optimal neurological clinical skills is lengthy and time-consuming, digital devices can represent cost-effective solutions that enhance the diagnosis and care of neurological patients in underserved regions of the world. Third, with the use of digital technology and new methods of analysis, detailed and subtle aspects of the patient’s bodily response become “visible” that cannot be perceived with the clinical eye. In this case, there can be an enhancement of diagnostic accuracy and a reshaping of the understanding of clinical manifestations of neurological diseases.

It seems astonishing that despite the triumphant advance of digital technology and new analysis methods for at least three decades, the “heart” of neurological diagnostics has remained almost completely untouched. One reason for this is probably the view of us neurologists that good NE can only take place in close personal contact between patient and professional. In our opinion, this argument does not speak against the implementation of new technology, as ideally it would practically not change the examination per se but would only record it and make more parameters available (Table 1). Another reason for not implementing digital technologies and new analysis strategies in NE could be data protection aspects. In our opinion, these are no more relevant than they are for already established examinations such as MRI and EEG. A compelling argument for the lack of implementation of digital NE is certainly that there is simply no suitable tool for this at present. There is no comprehensive, approved, validated, easy to use, easy to interpret in terms of extracted parameters, and adequately priced tool for a digitized version of a NE on the market that is accepted and reimbursed by health insurance companies.

**Table 1:** Problems and possible solutions for the implementation of the digital neurological exam

Problem identified	Possible solutions
Underestimating the potential of the physical examination	Technology can resignify the role of the physical examination in clinical practice and potentially increase the importance of this step of the medical consultation.
Lack of methodologically well-designed studies that support the implementation of the new technological device in the physical examination	Design studies with greater methodological rigour and fund research projects with a clear and well-defined line of research.
High complexity of the proposed digital instruments	Simplify existing technologies and involve the neurologist and the patient from the beginning of the development process.
High cost of technology	Encourage the development of as many digital tools as possible and sensitize the public sector
Lack of cost-effectiveness analysis of new technologies	Design and execute studies dedicated to evaluating the cost-effectiveness of the new methods proposed in comparison with the traditional physical examination.
Lack of a minimum comparison in terms of accuracy and precision between the digital assessment and traditional neurological tests	Evaluate the superiority, equivalence and non-inferiority of the digital device in comparison with specific tests of the neurological exam

In our view, this urgently needs to be developed, and many different professional groups need to be involved in the development. As it is the “heart” of the neurologist, we should play a central role in the development.
